# Joint Content Recommendation and Delivery in Mobile Wireless Networks with Outage Management

**DOI:** 10.3390/e20010064

**Published:** 2018-01-15

**Authors:** Yaodong Li, Lingyu Chen, Haibin Shi, Xuemin Hong, Jianghong Shi

**Affiliations:** 1 School of Information Science and Technology, Xiamen University, Xiamen 361005, China; 2 Key Lab of Underwater Acoustic Communication and Marine Information, Ministry of Education, Xiamen University, Xiamen 361005, China

**Keywords:** content recommendation, content delivery, outage probability, radio resource allocation

## Abstract

Personalized content retrieval service has become a major information service that consumes a large portion of mobile Internet traffic. Joint content recommendation and delivery is a promising design philosophy that could effectively improve the overall user experience with personalized content retrieval services. Existing research mostly focused on a push-type design paradigm called proactive caching, which, however, has multiple inherent drawbacks such as high device cost and low energy efficiency. This paper proposes a novel, interactive joint content recommendation and delivery system as an alternative to overcome the drawbacks of proactive caching systems. We present several optimal and heuristic algorithms for the proposed system and analyze the system performance in terms of user interest and transmission outage probability. Some theoretical performance bounds of the system are also derived. The effectiveness of the proposed system and algorithms is validated by simulation results.

## 1. Introduction

Due to the rapid proliferation of content-centric applications (e.g., social media websites) and broadband mobile communication networks, content-retrieval service has become a major service that consumes a large portion of traffic on the mobile Internet. Personalized content-retrieval service is a novel type of content-retrieval service that can use recommender technologies [1] to recommend content to users. As an effective means for users to acquire the most relevant information from a massive pool of content, personalized content-retrieval service has quickly gained popularity in recent years.

The personalized content retrieval service consists of two basic tasks: content recommendation and content delivery. The former task is in charge of predicting each user’s interest in a piece of content based on contextual information such as users’ historical preference, social relationships, mood, time, location, etc. [1,2,3,4,5]. The latter task is in charge of delivering the requested contents to users with quality-of-service (QoS) guarantee, which is further translated to throughput and delay requirements on the underlying communication links. From the users’ perspective, the quality-of-experience (QoE) of a personalized content retrieval service is related to both tasks: at the semantic level, recommended content should first appear attractive to the user; at the data communication level, the process of content download/access should be smooth enough to avoid user frustrations.

Traditionally, content recommendation and content delivery are considered as separate tasks carried out by different commercial entities. The former task is performed by content providers (CP) or over-the-top players (OTTs), while the latter by content delivery networks (CDNs) or Internet service providers (ISPs). Recent studies suggested that adapting a joint CP-CDN design can greatly help to improve the system performance and user experience. For example, in the context of the fixed Internet, it has been shown that CP-level intelligence can be used to improve the performance of traditional CDN networks [6]. In mobile networks, a content-format adaptive system was proposed in [7], which can adapt content formats according to wireless channel conditions. Among the various proposals, joint content recommendation and delivery has emerged as an important class of design that has attracted significant research interest.

The underlying philosophy of joint content recommendation and delivery is to exploit the domain of the content file size as a new design space. This is based on the fact that different contents with roughly the same level of user interest may vary dramatically in file size. Such diversity in content file sizes can be exploited to shape the traffic volume, thereby giving content delivery another degree of freedom for performance optimization. For example, a user may have a relatively lower interest in a web page that is ten-times smaller in size than a video clip. When the channel is congested, it is desirable to recommend the web page to avoid causing excessive delays. On the contrary, when the channel is clear, recommendation of the video clip is preferred. In this way, content recommendation can serve as a new mechanism of congestion avoidance in the communication network. This is particularly useful for mobile communication networks, whose capacity is severely limited by the available radio and infrastructure resources.

In the literature, most studies on joint content recommendation and delivery in mobile communication networks fall into a category of technologies called proactive caching [8,9,10], which can recommend, push and cache contents of interest to the user devices according to the status of communications links. Multiple issues such as energy efficiency [11,12], multicast support [13,14], heterogeneous networks [14] and adaptive traffic pricing [15,16] had been investigated. Although proactive caching technology can help to improve network performance via load balancing and enhance user QoE by reducing content access delay, it also has some inherent drawbacks. First, large caching space is required at the user device, which is not always available in practice. Second, even using the state-of-the-art content recommendation technologies, a significant portion of pushed content will not be viewed by the users due to user behavior uncertainties [17]. This will result in significant waste of network resource and energy consumption. Third, because a portion of pushed contents is unavoidably “invalid”, traffic pricing also becomes a problematic issue.

**Figure 1:**
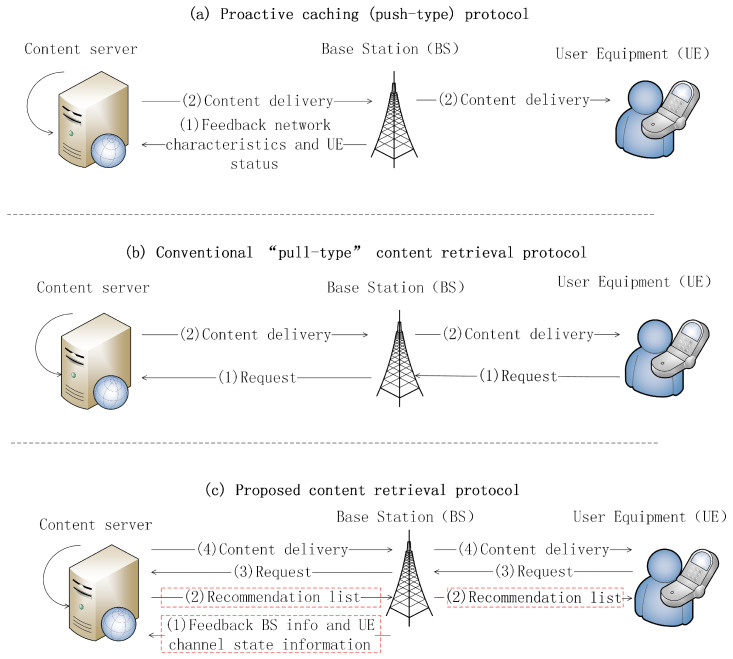
Illustration of the proactive caching protocol, the conventional pull-type protocol and the proposed content retrieval protocol.

Apart from the push-type protocols, a few novel “pull-type” content retrieval protocols were recently reported in the literature [18,19,20,21,22]. These studies mainly focused on how to use traffic pricing as an incentive to influence user behavior and achieve better performance in terms of load balancing [18,19,20] or load offloading [21,22]. These pull-type protocols proposed in [18,19,20,21,22], however, do not involve the procedure of content recommendation. Thus, we classify them as the “conventional pull-type protocols”. The typical signaling procedure of conventional pull-type protocols is illustrated in Figure 1b.

To overcome the drawbacks of proactive caching, this paper introduces a novel pull-type joint content recommendation and delivery design for personalized content-retrieval services. The contributions of our paper are as follows. First, an interactive, cross-layer-based joint content recommendation and delivery protocol is proposed. Second, based on the proposed protocol, optimal and low-complexity heuristic algorithms are given to jointly optimize content recommendation and radio resource allocation. Third, several theoretical bounds are derived to characterize the performance of the proposed system in a simple scenario. Fourth, the performance of the proposed algorithms is thoroughly compared via simulation in complex scenarios with realistic parameters. Simulation results show that the proposed system can achieve a good balance between maximizing user interest and minimizing transmission outage probability.

The remainder of this article is organized as follows. Section 2 introduces the system model and formulates the problem of joint content recommendation and delivery. The optimal and heuristic algorithms to solve the problem are proposed in Section 3 and Section 4, respectively. A novel performance evaluation framework is introduced in Section 5, followed by derivations of several theoretical performance bounds in Section 6. Section 7 presents simulation results. Finally, conclusions are drawn in Section 8.

## 2. System Model

### 2.1. Interactive Content Retrieval Protocol

In this paper, we propose a novel interactive content retrieval protocol for personalized multi-user content retrieval in mobile communication networks. As illustrated in Figure 1, the proposed protocol includes the following four steps: (1) The BS monitors the channel state information (CSI) of each active user and sends the CSI information to the content server; (2) The content server continuously runs a recommender algorithm and maintains a user interest matrix R (users are arranged in a column and contents in a row). Each element of R is a real number indicating a user’s interest in a piece of content. Based on R and the CSI information feedback, the server generates a list of recommended content for each user and sends the lists to users. The list is a small file that includes the title or short abstract of the contents; (3) Each user browses the recommended list and chooses interesting files from the list to download and view; (4) The chosen files are transmitted to users via a shared wireless channel. The basic idea of our protocol is to jointly consider user interest, channel condition and content file size in the content retrieval process, so that only files that are likely to be delivered in time will be recommended to users.

The proposed protocol is different from existing protocols. Compared with the “push-type” proactive caching protocols (e.g., [8,9]), our protocol will not start transmitting a file before an actual user request occurs. Therefore, it is a “pull-type” protocol and does not have the inherent drawbacks of proactive caching. Compared with conventional “pull-type” protocols (e.g., the hypertext transport protocol), our protocol is more advance in that it jointly considers the physical layer constraint (i.e., channel information) and application layer semantics (e.g., user interests) to manage the overall system performance. In this way, our protocol can not only give users better experiences in content retrieval, but also avoid network congestion and ensure better coexistence with other applications. The typical signaling procedures of the push-type protocol, conventional pull-type protocol and the proposed protocol are illustrated in Figure 1a–c, respectively. Moreover, the advantages and disadvantages of these three types of protocols are summarized in Table 1.

### 2.2. Scenario Description

The performance of the proposed protocol will be analyzed in a scenario described below. Without loss of generality, we consider a single cell with one base station (BS) and multiple users. The BS is connected to a content server, which stores a large set of contents. The number of users in the cell is denoted as *U*; the number of content files stored in the server is denoted as *F*. Let f(f=1,2,…,F) and u(u=1,2,…,U) be the indexes of the content file and user, respectively. The interest of the *u*-th user in the *f*-th file is represented by a real value parameter $r_{uf}\left(0\leq r_{uf}\right)$. The user interest matrix R is a U×F matrix, whose entries are taken from $r_{uf}$. We assume that by applying existing recommendation technologies [23,24,25,26], the user interest matrix R is known by the server in advance. The goal of the content retrieval system is to maximize the aggregated interests of recommended content files that can be delivered to users in time.

To proceed with our analysis, the following assumptions are made. (1) We assume that the size of a recommendation list file is much smaller than the size of the content files, so that transmission of the recommendation list (in Step 2 of the protocol) costs negligible time. (2) It is assumed that after browsing the recommended list, each user will click one and only one content file to view at a time instance. (3) We assume that the (slow fading) channel gains remain consistent in a recommendation cycle, i.e., the channel gains do not change during a single round of the four steps of the proposed protocol. (4) We consider an extreme case where user behaviors are synchronous, so that all users request a piece of content at the same time. This extreme case represents the worst case scenario because it is the most demanding for system capacity. Our subsequent analysis will focus on such a worst case scenario.

### 2.3. Wireless Transmission Model

An orthogonal frequency division multiplexing (OFDM)-based multi-user wireless transmission system is assumed. The number of OFDM subcarriers is denoted as *K*. Let us denote $\alpha_{uk}^{2}$ as the instantaneous channel gain between the BS and the *u*-th user on subcarrier *k* (k=1,2,…,K), $c_{uk}$ as the number of bits allocated to user *u* on subcarrier *k* and $p_{uk}$ as the transmit energy assigned to user *u* on subcarrier *k*. We have $p_{uk}=f\left(c_{uk}\right)/\alpha_{uk}^{2}$, where f(c) represents the transmit energy required for the subcarrier to reliably receive *c* bits per symbol when the channel gain is one. It is assumed that the channel gains follow an exponential distribution [27,28,29]. The total transmit power of the BS is denoted as $P_{T}$.

We consider a *M* quadrature amplitude modulation (*M*-QAM) system. It follows that $M=2^{c}$, where *c* is the number of bits carried by a QAM symbol. In this case, the bit error rate (BER) of the system is given by [30]:

(1) $$P_{e}\approx 4Q\left(\sqrt{\frac{d^{2}}{2N_{0}}}\right)$$

where *d* is the minimum distance between two points in the signal constellation, $N_{0}$ is the additive white Gaussian noise (AWGN) power spectral density and Q(·) is the Q-function [31]. The average carrying energy of an *M*-QAM signal is [30]:

(2) $$f\left(c\right)=\left(2^{c}-1\right)d^{2}/6.$$

Substituting Equation (1) into (2), we get [30]:

(3) $$f\left(c\right)=\frac{N_{0}}{3}{\left[Q^{-1}\left(\frac{P_{e}}{4}\right)\right]}^{2}\left(2^{c}-1\right).$$

This equation establishes the required received power f(c) as a function of bits per symbol *c* at a target BER $P_{e}$. Let us further define a binary variable $\rho_{uk}\in \left\{0,1\right\}$ to denote whether channel *k* is assigned to user *u*. When the channel is assigned, we have $\rho_{uk}=1$. The energy assigned to user *u* is:

(4) $$P_{u}=\sum_{k=1}^{K}p_{uk}*\rho_{uk}=\sum_{k=1}^{K}\frac{f(c_{uk})}{\alpha_{uk}^{2}}*\rho_{uk}$$

where $P_{u}$ is the energy required for user *u* to transmit $\sum_{k=1}^{K}c_{uk}*\rho_{uk}$ bits per symbol. Therefore, the total power allocated to user *u* is:

(5) $$P_{u}^{total}=B*\sum_{k=1}^{K}p_{uk}*\rho_{uk}$$

where *B* is the system bandwidth. Because the total power of the BS is constrained by $P_{T}$, we have:

(6) $$B*\sum_{u=1}^{U}\sum_{k=1}^{K}p_{uk}*\rho_{uk}\leq P_{T}.$$

Let us define $S_{u}$ as the transmit data rate assigned to user *u*, which is the sum of the number of bits in all subcarriers allocated to the user. It follows that:

(7) $$S_{u}=\sum_{k=1}^{K}c_{uk}*\rho_{uk}\;\left(\mathrm{bit}/\mathrm{symbol}\right).$$

Given the system bandwidth *B*, the bit rate of user *u* is approximately:

(8) $$R_{u}=S_{u}*B=\sum_{k=1}^{K}c_{uk}*\rho_{uk}*B\;\left(\mathrm{bit}/s\right).$$

### 2.4. Problem Formulation

We consider the decision problem of joint content recommendation and delivery at the content server. Apart from tracking the users’ interests on content files, the content server also periodically monitors the user and channel dynamics reported from the BS. Based on such information, a decision should be made to recommend to each user a list of *N* contents. To manage the user QoE, the recommendation algorithm should guarantee that in the worst case (when all users each request a piece of content from their lists at the same time), the system capacity is able to satisfy the user requests within a time constraint $T_{s}$.

At the system level, the service QoE is evaluated by two matrices. One is the sum user interests over recommended contents and the other is the outage probability in the worst case scenario. There is a tradeoff between these two matrices. As a result, the basic idea underpinning our problem formation is to maximize the sum user interests, under the condition that the outage probability is constrained by a predefined parameter. However, in practice, the exact outage probability is difficult to calculate. Therefore, as an indirect approach to outage management, we propose two alternative methods to constrain the sizes of recommended files according to the available channel capacity. The first approach constrains the maximum file size to give:

(9) $$\max_{f=1\cdot \cdot \cdot F}\left\{x_{uf}l_{f}\right\}\leq \delta *B*\sum_{k=1}^{K}c_{uk}*\rho_{uk}*T_{s}\;\forall u$$

where $x_{uf}\in \left\{0,1\right\}$ is the decision variable that takes binary values. When $x_{uf}$ is one, it means that content *f* is recommended to user *u*, otherwise $x_{uf}$ is zero. Here, parameter δ(1≤δ) is a parameter used to control the outage probability. When δ=1, this means that the recommendation is conservative in the sense that the maximum size of all *N* recommended files will not be greater than the estimated channel capacity allocated to the user. When δ is larger than one, this means that a certain outage is allowed in the system. The maximum file size constraint in (9) is intuitive, but nonlinear. To simplify the problem, it is desirable to have a linear constraint. To this end, we propose another constraint as follows:

(10) $$\frac{1}{N}\sum_{f=1}^{F}x_{uf}l_{f}\leq \delta *B*\sum_{k=1}^{K}c_{uk}*\rho_{uk}*T_{s}\;\forall u.$$

This constraint limits the average file size in the recommendation list instead of the maximum file size.

Applying the above constraints, the problem of joint content recommendation and delivery is formulated as:

(11) $$\begin{array}{cc} \mathrm{maximize} & \sum_{u=1}^{U}\sum_{f=1}^{F}r_{uf}x_{uf}\\ \mathrm{subject}\;\mathrm{to} & \mathrm{Equation}\;\;(9)\;\;\mathrm{or}\;\;(10)\\ & B*\sum_{u=1}^{U}\sum_{k=1}^{K}\frac{f(c_{uk})}{\alpha_{uk}^{2}}*\rho_{uk}\leq P_{T}\\ & \sum_{f=1}^{F}x_{uf}=N\\ & \sum_{u=1}^{U}\rho_{uk}\leq 1\\ & x_{uf}\in \left\{0,1\right\},\rho_{uk}\in \left\{0,1\right\}. \end{array}$$

In this problem formulation, the objective is to maximize the total user interest. The first constraint corresponds to the capacity outage control; the second constraint reflects the total BS transmit power limit $P_{T}$; the third constraint limits the number of recommended files to be *N*; the fourth constraint implies orthogonal subcarrier allocation. The decision variables in our problem are $x_{uf}$, $\rho_{uk}$ and $c_{uk}$. This means that the optimization is jointly performed over content recommendation and content delivery (i.e., channel/power/bit allocations in OFDM-based wireless communications systems). If we do not consider the aspects of content delivery, the above problem reduces to the traditional recommendation problem given by:

(12) $$\begin{array}{cc} \mathrm{maximize} & \sum_{u=1}^{U}\sum_{f=1}^{F}r_{uf}x_{uf}\\ \mathrm{subject}\;\mathrm{to} & \sum_{f=1}^{F}x_{uf}=N\\ & x_{uf}\in \left\{0,1\right\}. \end{array}$$

The traditional recommendation is able to recommend the most interested contents to users, but often at the cost of high capacity outage probability and hence degraded user experience. The solution of the traditional recommendation problem will be used as a performance benchmark in this paper.

## 3. Optimal Algorithm for Joint Content Recommendation and Delivery

The problem of joint content recommendation and delivery formulated in (11) is a nonlinear mixed integer programming problem, which is NP-hard. The optimal solution to this problem can be found by using the branch and bound algorithm [32,33,34], which is a general method for global optimization in nonconvex problems. If the feasible space is continuous, this algorithm can give a provable upper and lower bound on the (globally) optimal objective value and terminate with a certificate proving that the suboptimal point found is ϵ-suboptimal. For integer programming problems, the branch and bound algorithm can essentially search the entire feasible space to obtain the optimal solution. However, the complexity (or convergent rate) of the algorithm depends on the problem structure. In the worst case, the branch and bound method has an exponential complexity [35,36].

To improve the convergent rate of the branch and bound algorithm, a useful method is to linearize the problem and constraints. To this end, a linearization process is proposed as follows. First, let us define *C* as the maximum number of bits that can be transmitted in a QAM symbol. It follows that the feasible space of bit allocation is integer, i.e., $c_{uk}\in \left\{0,1,2,\ldots ,C\right\}$. Given $c_{uk}$, the required received power $f_{u}\left(c_{uk}\right)$ can be calculated as constants according to (3), i.e.,

(13) $$f_{u}\left(c_{uk}\right)\in \left\{0,f_{u}\left(1\right),f_{u}\left(2\right),\ldots ,f_{u}\left(C\right)\right\}.$$

Now, define a new variable [37]:

(14) $$\gamma_{ukc}=\left\{\begin{array}{cc} 1 & \mathrm{if}\;\rho_{\mathrm{uk}}=1\;\mathrm{and}\;c_{\mathrm{uk}}=c\\ 0 & \mathrm{otherwise} \end{array}\right.$$

It follows that we can rewrite $f_{u}\left(c_{uk}\right)$ and $\rho_{uk}$ as [37]:

(15) $$f_{u}\left(c_{uk}\right)=\sum_{c=0}^{C}\gamma_{ukc}f_{u}\left(c\right)$$

and:

(16) $$\rho_{uk}=\sum_{c=0}^{C}\gamma_{ukc}.$$

Substituting (15) and (16) into (9) and (10), we get:

(17) $$\max_{f=1\cdot \cdot \cdot F}\left\{x_{uf}l_{f}\right\}\leq \delta *B*\sum_{k=1}^{K}\sum_{c=0}^{C}c\gamma_{ukc}*T_{s}\;\forall u$$

and:

(18) $$\frac{1}{N}\sum_{f=1}^{F}x_{uf}l_{f}\leq \delta *B*\sum_{k=1}^{K}\sum_{c=0}^{C}c\gamma_{ukc}*T_{s}\;\forall u$$

for the maximum file size constraint and average file size constraint, respectively. Substituting Equations (15)–(18) into (11), we can transform the original optimization problem into:

(19) $$\begin{array}{cc} \mathrm{maximize} & \sum_{u=1}^{U}\sum_{f=1}^{F}r_{uf}x_{uf}\\ \mathrm{subject}\;\mathrm{to} & \mathrm{Equation}\;\;(17)\;\;\mathrm{or}\;\;(18)\\ & B*\sum_{u=1}^{U}\sum_{k=1}^{K}\sum_{c=0}^{C}\frac{f(c)}{\alpha_{uk}^{2}}*\gamma_{ukc}\leq P_{T}\\ & \sum_{f=1}^{F}x_{uf}=N\\ & \sum_{u=1}^{U}\sum_{c=0}^{C}\gamma_{ukc}\leq 1\\ & x_{uf}\in \left\{0,1\right\},\gamma_{ukc}\in \left\{0,1\right\}. \end{array}$$

In this new problem, the decision variables are $x_{uf}$ and $\gamma_{ukc}$. Simulations show that the linearization can significantly improve the convergence rate of the branch and bound algorithm. However, in the worst case, the algorithm complexity is still exponential (i.e., $O(2^{U^{2}FKC})$). Therefore, we will subsequently propose several heuristic algorithms to reduce the algorithm complexity.

## 4. Heuristic Algorithms for Joint Content Recommendation and Delivery

In this section, we propose several heuristic algorithms that divide the task of joint content recommendation and delivery into two steps. The first step is resource allocation, which aims to optimize multi-user radio resource allocation based on certain heuristics, for example to maximize the sum capacity or user fairness. After this step, each user will have a pre-allocated transmission capacity. The second step is content recommendation, which aims to maximize user interests based on the pre-allocated user capacity. We note that the above heuristic algorithms still try to jointly optimize content recommendation and delivery. The difference with the optimal algorithm is that in the optimal algorithm, content recommendation and radio resource allocation are jointly optimized, so that the radio resource allocated to a user is directly related to the user’s interest profile. In the proposed heuristic algorithms, radio resource allocation is based on other chosen criteria and is hence not directly related to the users’ interest profiles. In what follows, we will introduce the heuristic algorithms in detail and analyze their performance.

### 4.1. Radio Resource Allocation

Two different heuristic algorithms are proposed for radio resource allocation: the sum rate maximization algorithm and minimum rate maximization algorithm.

#### 4.1.1. Sum Rate Maximization

A straight-forward heuristic is to maximize the sum data rate of multi-user OFDM systems. In this case, the problem becomes a classic multi-user OFDM system rate adaptive optimization problem. The optimization problem is formulated as:

(20) $$\begin{array}{cc} \mathrm{maximize} & \sum_{u=1}^{U}\sum_{k=1}^{K}c_{uk}\rho_{uk}\\ \mathrm{subject}\;\mathrm{to} & B*\sum_{u=1}^{U}\sum_{k=1}^{K}\frac{f(c_{uk})}{\alpha_{uk}^{2}}*\rho_{uk}\leq P_{T}\\ & \sum_{u=1}^{U}\rho_{uk}\leq 1 \end{array}$$

where decision variables are bit allocation variables $c_{uk}$ and channel allocation variables $\rho_{uk}$. This problem is itself an NP-hard integer programming problem. To solve this problem effectively, an improved subcarrier and power adaptive allocation algorithm is proposed. The pseudo code of the algorithm is shown in Algorithm 1. The algorithm is a low complexity suboptimal algorithm, which includes two steps: subcarrier allocation and bit/power allocation. First, let us define a new variable:

(21) $$\Delta p_{uk}\left(c\right)=\frac{f(c+1)-f(c)}{\alpha_{uk}^{2}}$$

which indicates the extra power required when one more bit is allocated to user *u* on subcarrier *k*. This variable is used for our algorithm to allocate the channel and bits in a greedy fashion. To prevent the case where some users are not assigned with any bit, the algorithm initiates by allocating a carrier to each user. Once the bit allocation is completed, we can get the available transmission data rate of each user.

**Algorithm 1** Sum rate maximization algorithm.
**Input:** *U*, *K*, $P_{T}$, $\alpha_{uk}^{2}$
**Output:** $c_{u}$, $P_{u}$
1:(1) **Initialization**:  Let A={1,2,…,K} denote unassigned subcarrier sets; $P_{u}=0$ is the power allocated to user *u*; $c_{uk}=0$ represents the number of bits allocated on the subcarrier *k* allocated to the user *u*; $c_{u}=0$ indicates the total number of bits allocated to the user *u*; $A_{u}=⌀$ denotes the set of subcarriers assigned to the user *u*;  2:(2) **Carrier allocation**:  3:**for** u=1,2,…,U **do**  4:    find subcarrier *k* that minimizes $\Delta p_{uk}\left(0\right)$, and assign the subcarrier to the user *u*; meanwhile, $A_{u}=A_{u}\cup \left\{k\right\}$, A=A−{k}5:    **if** A=⌀ **then** break;6:**while** A≠⌀ **do**  7:    find the subcarrier *k* with the smallest $\Delta p_{uk}\left(0\right)$ in the set *A*, and assign them to the corresponding user *u*; meanwhile $A_{u}=A_{u}\cup \left\{k\right\}$, A=A−{k}8:(3) **Bit and power allocation**:   9:**while** $\sum_{u=1}^{U}p_{u}\leq P_{T}$ **do**  10:    Traverse the subcarriers corresponding to all the users; find the subcarrier *k* and the corresponding user *u* with the smallest $\Delta p_{uk}\left(c_{uk}\right)$, and allocate 1 bit of data to the subcarrier; meanwhile $c_{uk}=c_{uk}+1$, $P_{u}=P_{u}+\Delta p_{uk}\left(c\right)$, $c_{u}=c_{u}+1$  11:**return** $c_{u}$, $P_{u}$


We note that Algorithm 1 tends to allocate more bits and power to users with good channel conditions. Although this will maximize the sum capacity, it results in unfairness among users and does not necessarily give high total interest. For example, a user with poor channel condition may be allocated with a very low capacity and hence cannot support the transmission of large, but highly interested files. To overcome such a drawback and improve user fairness, the following algorithm is proposed as an alternative to Algorithm 1.

#### 4.1.2. Minimum Rate Maximization

Another useful heuristic is to maximize the minimum data rate among multiple users. This means that users with poorer channel conditions will have higher priorities in resource allocation and that the capacity allocated to users tends to be equal and fair. The optimization problem is a max-min problem formulated as:

(22) $$\begin{array}{cc} \max\min_{u=1\cdot \cdot \cdot U} & \sum_{k=1}^{K}c_{uk}\rho_{uk}\\ \mathrm{subject}\;\mathrm{to} & B*\sum_{u=1}^{U}\sum_{k=1}^{K}\frac{f(c_{uk})}{\alpha_{uk}^{2}}*\rho_{uk}\leq P_{T}\\ & \sum_{u=1}^{U}\rho_{uk}\leq 1. \end{array}$$

This problem can be effectively solved by a heuristic algorithm proposed as Algorithm 2.

**Algorithm 2** Minimum rate maximization algorithm.
**Input:** *U*, *K*, $P_{T}$, $\alpha_{uk}^{2}$
**Output:** $c_{u}$, $P_{u}$
1:(1) **Initialization**:  Let A={1,2,…,K} denote unassigned subcarrier sets; $P_{u}=0$ is the power allocated to user *u*; $c_{uk}=0$ represents the number of bits allocated on the subcarrier *k* allocated to the user *u*; $c_{u}=0$ indicates the total number of bits allocated to the user *u*; $A_{u}=⌀$ denotes the set of subcarriers assigned to the user *u*;2:(2) **Carrier allocation**:3:**while** A≠⌀ **do**4:    **for** u=1,2,…,U **do**5:        find the subcarrier *k* with the smallest $\Delta p_{uk}\left(0\right)$ in the set *A*, and assign them to the corresponding user *u*; meanwhile $A_{u}=A_{u}\cup \left\{k\right\}$, A=A−{k}  6:        **if** A=⌀ **then** break;7:(3) **Bit and power allocation**:  8:**while** $\sum_{u=1}^{U}p_{u}\leq P_{T}$ **do**  9:    **for** u=1,2,…,U **do**  10:        Traverse the subcarriers corresponding to the user *u*; find the subcarrier *k* with the smallest $\Delta p_{uk}\left(c_{uk}\right)$, and allocate 1 bit of data to the subcarrier; meanwhile $c_{uk}=c_{uk}+1$, $P_{u}=P_{u}+\Delta p_{uk}\left(c\right)$, $c_{u}=c_{u}+1$  11:        **if** $\sum_{u=1}^{U}p_{u}>P_{T}$ **then** break;12:**return** $c_{u}$, $P_{u}$


Once the pre-allocation of radio resource is completed, the data rate of user *u* can be calculated as:

(23) $$c_{u}=\sum_{k=1}^{K}c_{uk}\rho_{uk}\;(\mathrm{bit}/\mathrm{symbol}).$$

The bit rate assigned to the *u*-th user is approximately:

(24) $$R_{u}=c_{u}*B=\sum_{k=1}^{K}c_{uk}\rho_{uk}*B\;(\mathrm{bit}/s).$$

### 4.2. Content Recommendation

Based on the pre-allocated user capacity, we can formulate two content recommendation problems as follows.

#### 4.2.1. Maximum File Size Constraint

(25) $$\begin{array}{cc} \mathrm{maximize} & \sum_{u=1}^{U}\sum_{f=1}^{F}r_{uf}x_{uf}\\ \mathrm{subject}\;\mathrm{to} & l_{max}^{u}=\max_{f=1\cdot \cdot \cdot F}\left\{x_{uf}l_{f}\right\}\leq \delta *R_{u}*T_{s}\;\forall u\\ & \sum_{f=1}^{F}x_{uf}=N. \end{array}$$

In this problem, maximizing the sum interest is equivalent to maximizing each user’s interest because the capacities of users are already fixed and decoupled. The optimal algorithm that solves this problem runs as follows: For each user, we exclude contents whose size exceeds the data rate $R_{u}$ and then recommend the top *N* contents with the highest interests in the remaining content. The minimum heap algorithm (time complexity O(FlogN)) or partial sorting algorithm (time complexity O(FN)) can be used here.

#### 4.2.2. Average File Size Constraint

(26) $$\begin{array}{cc} \mathrm{maximize} & \sum_{u=1}^{U}\sum_{f=1}^{F}r_{uf}x_{uf}\\ \mathrm{subject}\;\mathrm{to} & \frac{1}{N}\sum_{f=1}^{F}x_{uf}l_{f}\leq \delta *R_{u}*T_{s}\;\forall u\\ & \sum_{f=1}^{F}x_{uf}=N. \end{array}$$

Similarly, this problem can be decoupled and solved with respect to each user. For each user, the problem becomes a two-dimensional cost knapsack problem [38], which could be solved by dynamic programming. Define g(f,l,j) to be the maximum interest that can be attained with the file size less than or equal to *l* and the number of contents less than or equal to *j* using contents up to *f* (first *f* contents). Then, the state transition equation is:

(27) $$g\left(f,l,j\right)=\max\left\{g\left(f-1,l,j\right),g\left(f-1,l-l_{f},j-j_{f}\right)+r_{uf}\right\}$$

where $l_{f}$ represents the size of content *f* and $j_{f}$ always equals one, which means that one content is recommended. That is, when it is decided whether or not to put the *f*-th content, we need to compare the interest in putting the *f*-th content and the interest in not putting the *f*-th content.

### 4.3. Complexity of Heuristic Algorithms

Let us first consider the time complexity of the two algorithms in resource allocation. Recall that *K* is the number of OFDM subcarriers and *U* is the number of users. For the sum rate maximization algorithm (i.e., Algorithm 1), the complexity is O(UK) in the carrier allocation step and $O(P_{T}K)$ at the bit allocation step. For the minimum rate maximization algorithm (i.e., Algorithm 2), the time complexity is $O(K^{2})$ in the carrier allocation step and $O(P_{T}\frac{K}{U})$ in the bit allocation step. In practice, *K* is typically much greater than *U*, so that Algorithm 1 tends to have a lower complexity compared with Algorithm 2.

Let us now consider the time complexity of the content recommendation algorithms. For the average file size constraint, the time complexity of the two-dimensional cost knapsack algorithm is $O(\delta FRN^{2})$, where *R* is the data rate assigned to the user. Here, *R* is related to power $P_{T}$, channel number *K* and user number *U*. Considering a total of *U* users, the total time complexity is $O(\delta UFRN^{2})$. For the problem with maximum file size constraint, the complexity of the algorithm is O(UFN).

We can see that the proposed heuristic algorithms can reduce the exponential complexity of the optimal algorithm to polynomial complexities as the problem size scales. However, the reduced complexity comes at a cost of degraded performance. For example, in a special case where a user with good condition is only interested in small files, the heuristic algorithm may assign a large amount of radio resource to this user to maximize the overall capacity. However, much of the capacity allocated to this user is unnecessary and wasted. In what follows, an analytical framework will be introduced to evaluate the performance of the proposed algorithms.

## 5. Performance Evaluation Framework

To evaluate the performance of joint content recommendation and delivery algorithms, we consider two major metrics: total user interest and transmission outage probability. The first metric is related to the effectiveness of content recommendation, while the second metric is related to the reliability of content delivery. The total user interest is given by:

(28) $$I_{total}=\sum_{u=1}^{U}\sum_{f=1}^{F}r_{uf}x_{uf}.$$

The transmission outage is defined as the probability that when each user randomly requests content from the recommended list (with *N* contents), the BS cannot deliver the request contents within a time constraint $T_{s}$. An outage event can be defined with respect to the following mathematical problem:

(29) $$\begin{array}{cc} & \mathrm{Find}\;c_{uk},\rho_{uk}\\ \mathrm{subject}\;\mathrm{to} & l_{i}^{u}\leq B*\sum_{k=1}^{K}c_{uk}*\rho_{uk}*T_{s}\;\forall u\\ & B*\sum_{u=1}^{U}\sum_{k=1}^{K}\frac{f(c_{uk})}{\alpha_{uk}^{2}}*\rho_{uk}\leq P_{T}\\ & \sum_{u=1}^{U}\rho_{uk}\leq 1\\ & x_{uf}\in \left\{0,1\right\},\rho_{uk}\in \left\{0,1\right\} \end{array}$$

where $l_{i}^{u}$ represents the size of the file *i* requested by user *u*. Because the file should be delivered within a time constraint $T_{s}$, it is directly related to the instantaneous data rate requirement of user *u*. The problem is whether there exist any feasible channel and power allocation policy (i.e., $c_{uk}$ and $\rho_{uk}$) that can satisfy the instantaneous data rate requirements of all users. If the above problem is solvable, this means that content delivery is successful; otherwise, a transmission outage occurs. We note that the above outage metric is defined at the system level by considering multiple users.

### Approximation of Outage Probability

Unfortunately, evaluating the system outage according to its real definition in Equation (29) is not only mathematically intractable, but also computationally challenging. To facilitate our performance evaluation, we propose a method that can approximately estimate the transmission outage according to the following equation:

(30) $$\eta =P\left(\sum_{u=1}^{U}l_{i}^{u}>C_{sys}*T_{s}\approx \sum_{u=1}^{U}R_{u}*T_{s}\right).$$

Here, the outage probability η is defined as the probability that the overall requested file size is greater than the instantaneous system capacity $C_{sys}$. This approximation essentially neglects the channel diversity of multiple users and uses a lump-sum capacity $C_{sys}$ to capture the resource limitation of the system. There exist many methods to calculate the lump-sum capacity $C_{sys}$. Without loss of generality, we propose to calculate $C_{sys}$ as the sum capacity of a system that adopts the same radio resource allocation strategy introduced in Section 4.1. In other words, the sum capacity obtained in the capacity estimation phase will be used as the lump-sum system capacity for outage estimation.

Now, we proceed to investigate fast algorithms to evaluate the system outage according to the definition in Equation (30). Once the user channels are known, $C_{sys}$ can be calculated. The challenge is to consider all possibilities of the sum sizes of requested content files. Let us consider *U* users each selecting a file from a list of *N* items; the feasible space of the users’ request vector has an exponential size of $N^{U}$. Although we could use the backtracking algorithm to enumerate all the possibilities [39], the computational complexity is too high. To this end, we further propose a fast method for outage estimation, as explained in Algorithm 3.

**Algorithm 3** The algorithm for outage estimation.1:Run the model *n* times, and get n∗N∗U recommended contents;2:Calculate the frequency of the file size of the above contents, then the PDF of file size in each user’s recommended list could be obtained;3:Assume that all users are independent of each other. Then, the joint PDF of sum file size of *U* users is the U−1 convolution of the above PDF;4:With the joint PDF of the sum file size of *U* users, the joint CDF could be obtained. Then, the outage is $1-CDF(C_{sys})$.


In Algorithm 3, we assume that all users are independent of each other. Hence, the outage can be evaluated using statistical methods.

**Figure 2:**
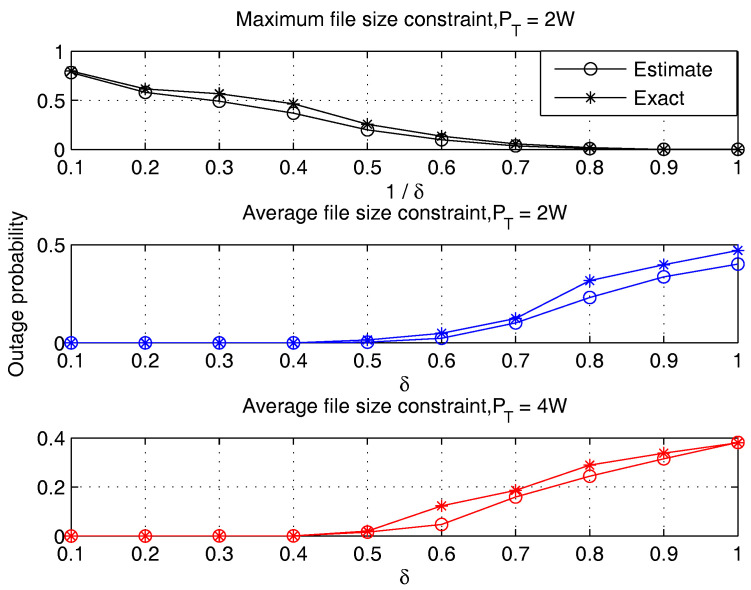
Comparison of the exact outage probability obtained by Monte Carlo simulations and the estimated outage probability calculated by Algorithm 3 (the file size distribution is subject to Lognorm(10,1), U=5, F=500, N=50, K=256).

The accuracy of our outage approximation method is evaluated via Monte Carlo simulations. In each simulation, a user request profile and user channel gains are randomly generated. The exact value of outage probability is obtained by solving a large incident of the problem defined in (29) and calculating the empirical probability. The approximated outage is obtained according to Algorithm 3. Figure 2 compares the exact and approximated outage in different settings. We can see that the estimated outage makes a fairly good approximation to the exact outage curve.

## 6. Theoretical Performance Bounds with Simple Models

This section aims to characterize some theoretical performance bounds of the joint content recommendation and delivery system. For tractability, we assume a simple scenario with simplified models. More specifically, it is assumed that the user interests in different contents follow a uniform distribution in [a,a+h] (e.g., rating scores uniformly distributed from 1–10), and all users’ interest profiles are independent. In addition, it is assumed that content file sizes also follow a uniform distribution in [b,b+g].

### 6.1. Upper Limit of Mean User Interests

We first evaluate the upper limit of mean user interests. Given that the users’ interest is uniformly distributed, the total interest in the highest *N* content scores for each user is:

(31) $$S=X_{\left(F+1-N\right)}+X_{\left(F+2-N\right)}+\ldots +X_{\left(F\right)}$$

where $X_{\left(i\right)}$ represents the *i*-th order statistics. According to [40,41,42], the PDF of the *i*-th order statistics of the standard uniform distribution is:

(32) $$f_{i}\left(x\right)=\frac{F!}{(i-1)!(F-i)!}x^{i-1}{\left(1-x\right)}^{F-i},0\leq x\leq 1.$$

That is, $X_{\left(i\right)}$ obeys the beta distribution with parameters *i* and F+1−i, where *F* is the total number of contents.

Given that $X=(X_{1},X_{2},\ldots ,X_{F})$ is a uniform distribution in [a,a+h], where a∈R, h∈(0,∞), then for i∈{1,2,…,F}, $X_{\left(i\right)}$ obeys a beta distribution with left parameter *i*, right parameter F−i+1, position parameter *a* and scale parameter *h*. In particular, we have [42]:

(33) $$E\left(X_{\left(i\right)}\right)=a+h\frac{i}{F+1}$$

(34) $$\mathrm{var}\left(X_{\left(i\right)}\right)=h^{2}\frac{i(F-i+1)}{{\left(F+1\right)}^{2}\left(F+2\right)}.$$

It follows that the mean of *S* is given by:

(35) $$\begin{array}{cc} E(S;F;N) & =E(X_{\left(F+1-N\right)}+X_{\left(F+2-N\right)}+\ldots +X_{\left(F\right)})\\ & =E\left(X_{\left(F+1-N\right)}\right)+E\left(X_{\left(F+2-N\right)}\right)+\ldots +E\left(X_{\left(F\right)}\right)\\ & =\left(a+h\frac{F+1-N}{F+1}\right)+\ldots +\left(a+h\frac{F}{F+1}\right)\\ & =Na+\frac{h}{F+1}\frac{(F+1-N+F)F}{2}\\ & =Na+\frac{Nh(2F+1-N)}{2(F+1)}. \end{array}$$

Here, E(S;F;N) is the total interest shown on the recommended list of an individual user. If there are *U* users, the total interest becomes:

(36) $$I_{total}^{est}=U\cdot E\left(S;F;N\right).$$

### 6.2. Upper Limit of Mean Outage

Let us assume that the number of files is *F*, and the file size is subject to a uniform distribution on [b,b+g]. We further assume that user’s interest is independent of the file size. In this case, each user’s recommended list is an independent sample on the content set *L*.

According to [43,44], we can see that if $X_{i}$ is uniformly distributed on $\left[0,g_{i}\right]$, the PDF of $S_{X}=X_{1}+X_{2}+\ldots +X_{U}$ is:

(37) fSX(x;U;gi)=1AU(U−1)!{xU−1+∑k=1U(−1)kUk[(x−∑l=1kgl)+]U−1}

where $A_{U}=\prod_{k=1}^{U}g_{k}$, $x_{+}=\max\left(0,x\right)$. If $Y_{i}$ follows a uniform distribution on $\left[b_{i},b_{i}+g_{i}\right]$, $S_{Y}=\sum_{i=1}^{U}Y_{i}=\sum_{i=1}^{U}x_{i}+\sum_{i=1}^{U}b_{i}$, and the PDF of $S_{Y}$ is given by:

(38) $$f_{S_{Y}}\left(s;U;b_{i};g_{i}\right)=f_{S_{X}}\left(\sum_{i=1}^{U}Y_{i}-\sum_{i=1}^{U}b_{i};U;g_{i}\right).$$

Correspondingly, the CDF of $S_{Y}$ is:

(39) $$F_{S_{Y}}\left(s;U;b_{i};g_{i}\right)=\int_{-\infty }^{s}f_{S_{Y}}\left(t;U;b_{i};g_{i}\right)\;dt.$$

When the system capacity $C_{sys}$ is given, the outage is estimated as:

(40) $$\hat{\eta }=1-F_{S_{Y}}\left(C_{sys}*T_{s};U;b_{i};g_{i}\right).$$

**Figure 3:**
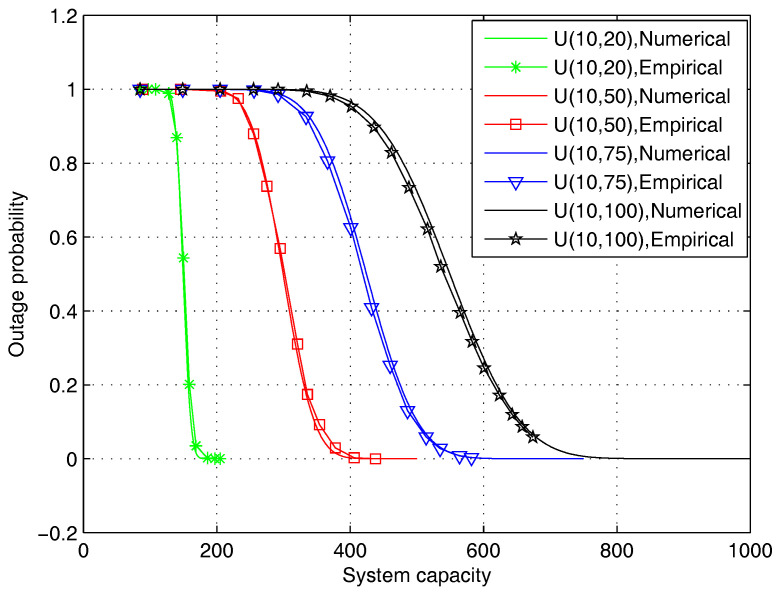
Comparison of the empirical value and the numerical value of the upper limit of outage, where U(x,y) represents a uniform distribution in [x,y].

In Figure 3, we compare the numerical CDF calculated by Equation (40) with the empirical CDF obtained via Monte Carlo simulations. The numbers of contents and users are set to be 500 and 10, respectively, and the length of the recommendation list is set to be 50. It can be observed that the numerical and empirical CDFs agree well in all cases with different file size distributions. This validates the correctness of Equation (40).

### 6.3. Upper Limit of Interest at Zero Outage

We assume that the file distribution follows a uniform distribution in [b,b+g]. Then, if the system capacity is $C_{sys}$ and the outage is required to be zero, the file size in each user’s recommended list should be limited to the range $\left[b,\frac{C_{sys}}{U}\right]$, which accounts for the ratio of the original interval [b,b+g] to:

(41) $$\varphi =\frac{\frac{C_{sys}}{U}-b}{g}=\frac{C_{sys}-Ub}{Ug}.$$

Because the file size is uniformly distributed, in the case where the system capacity is $C_{sys}$ and the outage is zero, the number of optional contents is approximately:

(42) $$\tilde{F}=F*\varphi .$$

Given the number of contents $\tilde{F}$, we can obtain the total interest when the outage is zero according to (36):

(43) $$I_{total}^{0}=U\cdot E\left(S;\tilde{F};N\right).$$

### 6.4. Validation of the Theoretical Bounds

In this subsection, simulations are performed to validate the theoretical bounds derived above. The parameters are set as follows: the user interest follows a uniform distribution in [1,10]; the file size follows a uniform distribution in [1,50]; the number of users U=10; the number of contents F=500; the length of the recommended list for each user N=50; and the system capacity is 188.

**Figure 4:**
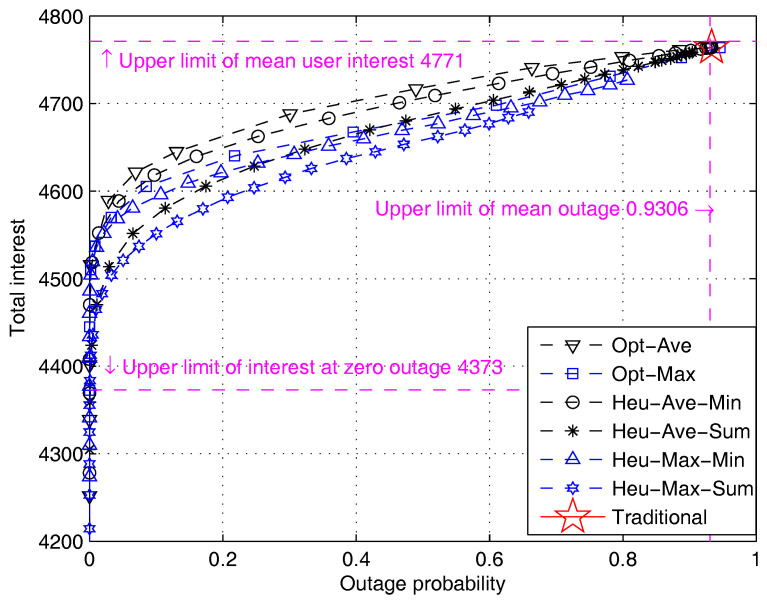
Performance comparison of different algorithms and theoretical performance bounds (simple model). Opt, optimal; Heu, heuristic.

Figure 4 shows the total user interest as a function of system outage when different algorithms proposed in Section 4 are applied. The performance tradeoff curves are obtained by adjusting the value of parameter σ in the algorithms. The theoretical bounds are also calculated and shown. The upper limit of interest is shown to be 4771 according to Equation (36); the upper limit of outage is shown to be 0.9306 according to Equation (40); and the upper limit of interest at zero outage is 4373. The point at (0.9306,4771) shows the performance benchmark of traditional recommendation algorithm, in which both the user interest and transmission outage reach the maximum. We can see that the three theoretical bounds derived above form a square area, which well characterizes the performance tradeoff region of the proposed algorithms.

## 7. Performance Evaluation with Realistic Models

This section aims to thoroughly evaluate the performance of the proposed algorithms in realistic scenarios. Some realistic models are first introduced, followed by performance comparisons and discussions.

### 7.1. Realistic Models

In reality, the content file size and user interest do not follow simple uniform distributions. Measurements showed that the file size is generally subject to a power law distribution [45] or a lognormal distribution [46,47,48,49,50]. In this paper, we assume that the file size follows a lognormal distribution. As for the distribution of aggregated/sum user interests on different contents, existing literature suggest that it generally follows a power law distribution [51] or Zipf distribution [52]. We adapt the widely-accepted Zipf distribution in this paper. Moreover, multiple users have different interest in a particular piece of content. The interest distribution among multiple users can be modeled by a normal distribution [53], U-shaped (or J-shaped) distribution [54,55], Beta distribution [56] or Levy alpha-stable distribution [57]. In this paper, the normal distribution is adopted as the multiuser interest model by default. Parameter values adopted for our simulation in this section are summarized in Table 2.

### 7.2. Simulation Results and Discussions

According to our discussions in Section 3 and Section 4, the performance of the following seven different algorithms will be evaluated: (1) the optimal algorithm with maximum file size constraint (Opt-Max); (2) the optimal algorithm with average file size constraint (Opt-Ave); (3) the heuristic algorithm with maximum file size constraint and sum rate maximization (Heu-Max-Sum); (4) the heuristic algorithm with maximum file size constraint and minimum rate maximization (Heu-Max-Min); (5) the heuristic algorithm with average file size constraint and sum rate maximization (Heu-Ave-Sum); (6) the heuristic algorithm with average file size constraint and minimum rate maximization (Heu-Ave-Min), and (7) the traditional “pull-type” content retrieval algorithm without outage management (traditional). The tradeoff between total user interest and outage probability will be used as the performance evaluation framework.

**Figure 5:**
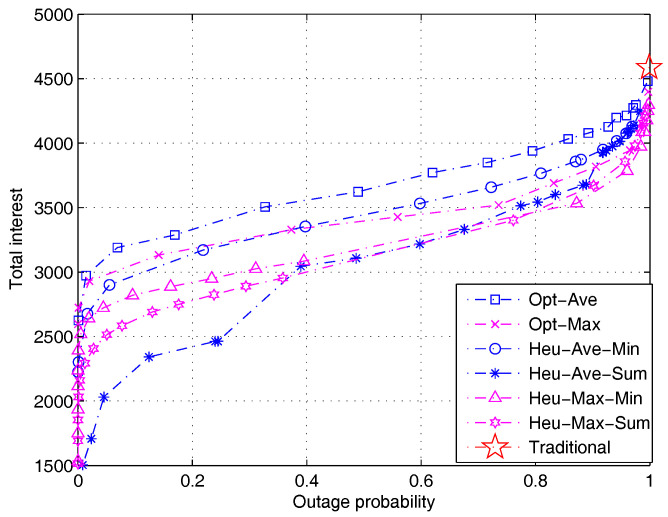
Performance comparison of different algorithms (file size distribution is subject to Lognorm(9.357,1.318), $P_{T}=0.5$ W).

Figure 5 compares the performance of all seven algorithms. The performance of the traditional “pull-type” content retrieval algorithm is characterized by a single point (marked by the star sign). This single point represents an extreme case where the system yields the highest user interest at the cost of the largest outage probability. We note that the traditional algorithm does not offer the flexibility to adjust the user interest or outage probability. In contrast, the performance of our algorithm is characterized by a smooth curve, in which user interest can be flexibly traded for outage probability. It is interesting to see that a small reduction in the user interest (e.g., a 25 percent reduction) can greatly reduce the outage probability by almost 90 percent. Such a capability to flexibly manage the outage probability is a major advantage of our algorithm compared with the traditional pull-type algorithm. The flexibility offered by our protocol essentially comes from exploiting the content diversity as a new degree of freedom.

Figure 5 also shows the performance comparison of different algorithms. Let us first compare the two optimal algorithms. It is observed that the Opt-Ave algorithm outperforms the Opt-Max algorithm. Moreover, the Opt-Ave algorithm tends to have a lower computational complexity than the Opt-Max algorithm because it solves a strictly linear programming problem. As a result, we conclude that the Opt-Ave algorithm has a better performance. As for the heuristic algorithms, it can be seen that the two algorithms adapting minimum rate maximization yield better performance than the two algorithms adapting sum rate maximization. This suggests that it is beneficial to allocate capacity evenly among users. It is shown that the best heuristic algorithms can achieve 80 percent of the optimal performance in the worst case. The performance loss is acceptable and is traded for much lower computational complexity.

**Figure 6:**
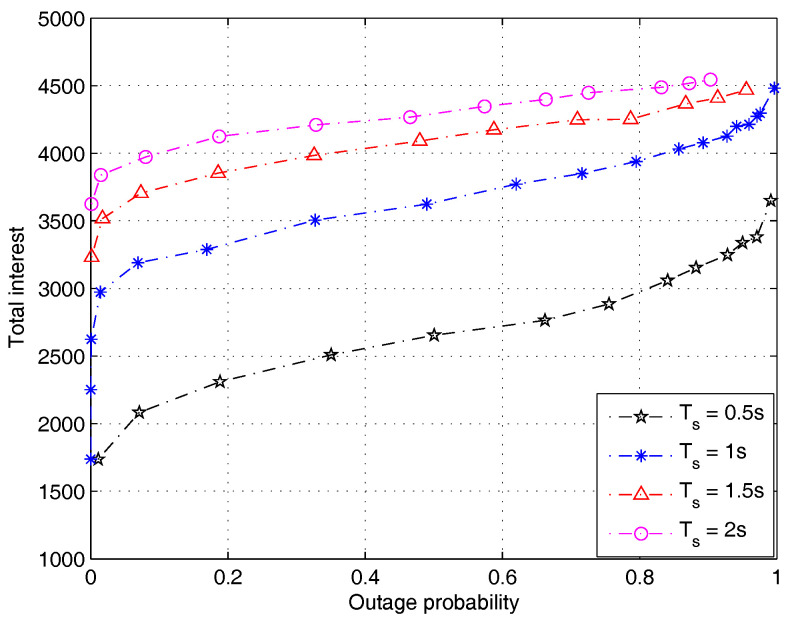
The effect of time constraint $T_{s}$ on the performance (the file size distribution is subject to Lognorm(9.357,1.318), $P_{T}=0.5$ W).

Figure 6 investigates the impact of time constraint $T_{s}$ on the system performance. The time constraint represents the maximum allowable delay for a user to pull a content file from a BS. It can be observed in Figure 6 that a decreased $T_{s}$ leads to a reduced user interest. This indicates the conflicting objectives of maximizing the user interest and minimizing the content access delay, where both objectives are desirable for enhancing user experience. In practice, a proper balance should be sought by setting a proper value for $T_{s}$. We note that in conventional pull-type protocols, the access delay is generally random and out of control. Therefore, the ability to manage access delay by adjusting $T_{s}$ is also a major advantage of our protocol compared with conventional pull-type protocols.

**Figure 7:**
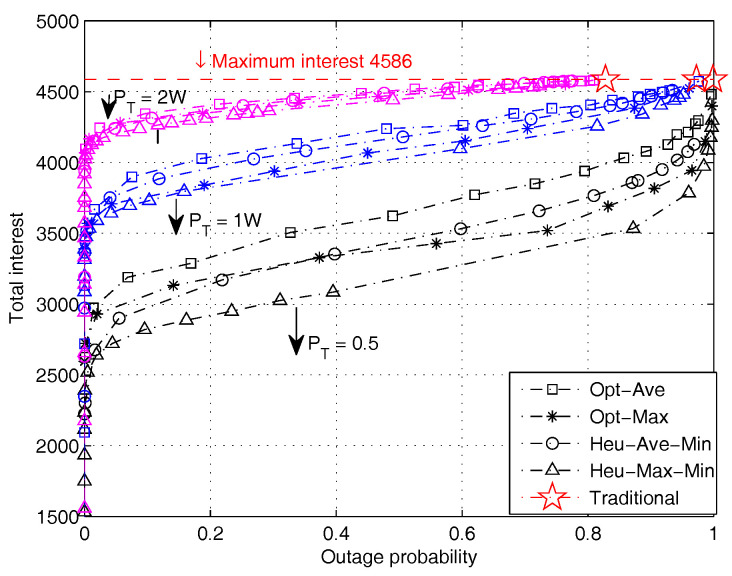
Performance comparison of different algorithms with varying power constraint $P_{T}$ (file size distribution is subject to Lognorm(9.357,1.318)).

In Figure 7, we demonstrate the impact of the total power constraint on the interest-outage tradeoff performance. The two optimal algorithms and the two heuristic algorithms adapting minimum rate maximization are simulated. It is observed that the overall user interest increases with increasing power and outage until it researches the theoretical upper bound, which is 4586 in this particular case. Moreover, when the power increases, the performance gaps between optimal and heuristic algorithms reduce. It is interesting to see that at relatively high power values, a fairly good performance can be achieved even when the outage is very small. For example, at $P_{T}=2$ W, nearly 90 percent of the optimal performance can be achieved at an outage of 0.05. This implies that sufficient power supply (relative to the file size characteristics) can ensure that the system operates in a desirable state with high performance and low outage.

**Figure 8:**
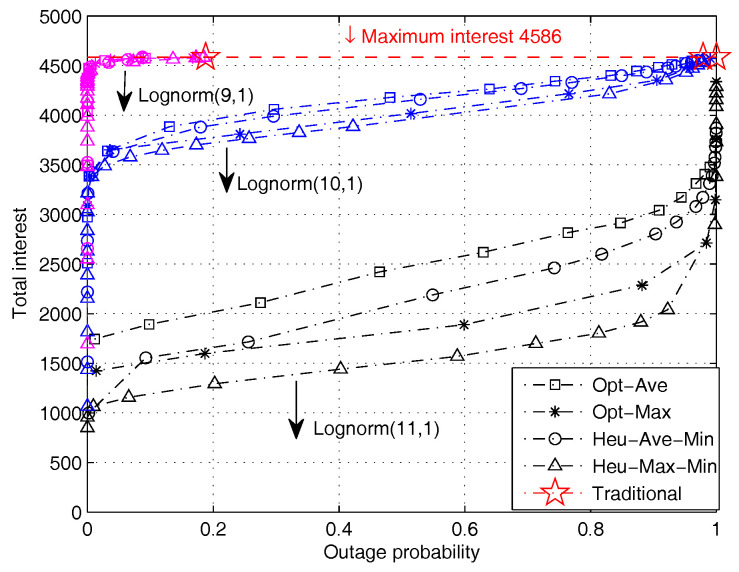
Performance comparison of different algorithms with varying file size distributions ($P_{T}=2$ W).

Figure 8 illustrates the impact of file size distribution on the system performance. It can be seen that the impact of reducing the average file size is very similar to the impact of increasing the power constraint. When the file size is small, the performance quickly approaches the optimal even at a low outage probability. This observation reinforces our previous remarks that power constraint and file size are two sides of the same coin and should be jointly considered when designing a system.

**Figure 9:**
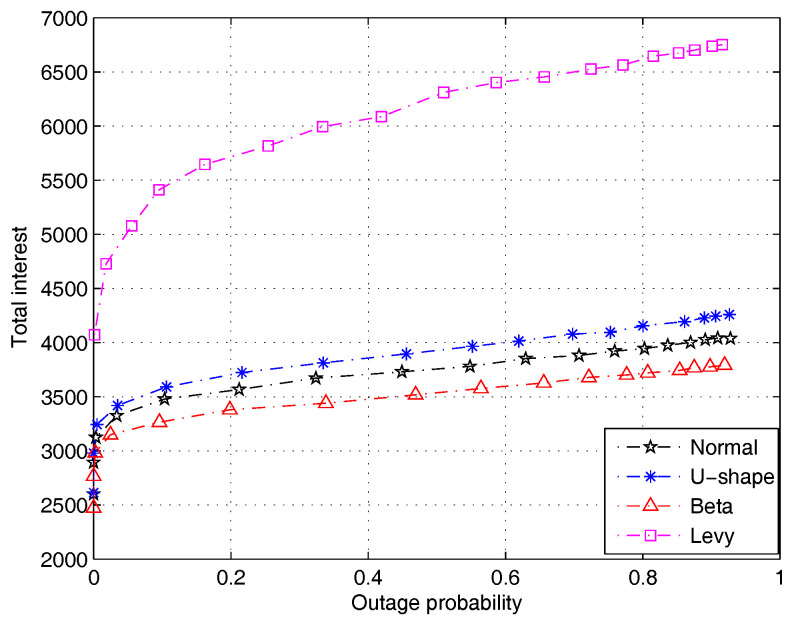
Performance comparison with different distributions of individual user’s interest in a piece of content across multiple users (file size distribution is subject to Lognorm(9.357,1.318), $P_{T}=1$ W).

Finally, Figure 9 compares the system performance when the distribution of individual user’s interest in a piece of content across multiple users has different forms. We simulate all four types of distributions reported in the literature. We can see that the normal, U-shaped and Beta distributions yield similar performance. However, the Levy distribution yields a much better performance in terms of the total user interest. This is because the former three distributions are balanced and the interest in a content file tends to spread across many users. On the contrary, the Levy distribution is a heavy-tailed distribution, so that the interest in a content file tends to concentrate with a few users. In this case, higher total user interest can be obtained by satisfying a few users that have very high interest.

## 8. Conclusions

In this paper, we have proposed a novel design for personal content retrieval systems to jointly optimize content recommendation and content delivery. Optimal algorithms with exponential complexities have been introduced to solve the joint optimization problem. A linearization technique has been proposed to reduce the computational complexity of the optimal algorithms. Moreover, several heuristic algorithms have been presented to tackle the joint optimization problem with polynomial complexity. The fundamental performance of the proposed system has been characterized by theoretical bounds and evaluated via simulations. Theoretical and simulation results have shown that the proposed system has the potential to achieve both high user interest and low transmission outage probability. Moreover, it has been demonstrated that the best performing heuristic algorithm can well approximate the optimal performance of the system. We conclude that the proposed system can effectively balance the conflicting goals of maximizing user interest and minimizing transmission outage; hence, it is a promising design paradigm for personalized content retrieval systems.

## Figures and Tables

**Table 1:** Pros and cons of the three types of protocols.

	Pros	Cons
Proactive caching protocol	1. Possibly less time delay.	1. Require large cache space;
		2. Low resource efficiency (due to invalid transmission);
		3. Difficult traffic pricing.
Conventional pull-type protocol	1. High resource efficiency;	Uncontrollable content access delay; Uncontrollable congestion probability.
	2. Small cache space and easy traffic pricing;	
	3. High user interest.	
Proposed content retrieval protocol	1. High resource efficiency;	1. User interest may be compromised.
	2. Small cache space and easy pricing;	
	3. Controllable content access delay;	
	4. Controllable congestion probability.	

**Table 2:** Simulation parameters.

Simulation Parameter	Parameter Value
Number of users *U*	10
Number of contents *F*	500
Number of channels *K*	256
Recommended form length *N*	50
Time slot $T_{s}$	1 s
System bandwidth *B*	10 MHz
Noise power spectral density $N_{0}$	−174 dBm/Hz [58]
Bit error rate BER	1 × 10${}^{-4}$ [37]
Macrocell path loss model	128.1 + 37.6$\log_{10}d$ (*d* in km) [58]
Inter-site distance *d*	330 m
Channel gain $\alpha_{uk}^{2}$	Exponential distribution of parameter 1
2* File distribution *L*	Logarithmic normal distribution with location parameter of 9.357 and scale parameter of 1.318 [47]
3*Interest matrix R	Zipf distribution with parameter 1 [52]; Truncated Gaussian distribution between 1 and 5 with a mean of 3 and a variance of 2 [53].
